# The Pivotal Role of NF-κB in Glioblastoma: Mechanisms of Activation and Therapeutic Implications

**DOI:** 10.3390/ijms26167883

**Published:** 2025-08-15

**Authors:** Vanajothi Ramar, Shanchun Guo, Guangdi Wang, Mingli Liu

**Affiliations:** 1 Department of Microbiology, Biochemistry & Immunology, Morehouse School of Medicine, Atlanta, GA 30310, USAmliu@msm.edu (M.L.); 2 RCMI Cancer Research Center and Department of Chemistry, Xavier University, 1 Drexel Dr, New Orleans, LA 70125, USA; gwang@xula.edu

**Keywords:** glioma, NF-κB, glioma stem cells, signaling pathway, drug target

## Abstract

Glioblastoma multiforme (GBM) is the most aggressive and lethal primary brain tumor in adults, characterized by high intratumoral heterogeneity, therapy resistance, and poor prognosis. Nuclear factor-κB (NF-κB) signaling plays a pivotal role in GBM pathogenesis by promoting proliferation, invasion, inflammation, immune evasion, and treatment resistance. This review provides a comprehensive overview of canonical and non-canonical NF-κB signaling pathways and their molecular mechanisms in GBM, with a focus on their regulation in glioma stem-like cells (GSCs), interactions with key oncogenic factors (including STAT3, FOSL1, and TRPM7), and roles in maintaining tumor stemness, metabolic adaptation, and angiogenesis. We further discuss the reciprocal regulatory dynamics between NF-κB and non-coding RNAs (ncRNAs), particularly microRNAs, highlighting novel ncRNA-mediated epigenetic switches that shape GBM cell plasticity and subtype specification. Additionally, we examine the influence of NF-κB in modulating the tumor microenvironment (TME), where it orchestrates pro-tumorigenic cytokine production, immune cell reprogramming, and stromal remodeling. Finally, we review current NF-κB-targeting therapeutic strategies in GBM, including clinical trial data on small-molecule inhibitors and combinatorial approaches. Understanding the multifaceted roles of NF-κB in GBM offers new insights into targeted therapies aimed at disrupting tumor-promoting circuits within both cancer cells and the TME.

## 1. Introduction

Glioblastoma (GBM) is the predominant form of primary brain tumor and the most aggressive variant in the adult nervous system, characterized by a dismal prognosis. The relative survival rate of GBM is approximately 4.5%, with individuals surviving five years post-diagnosis. GBM is classified into four histological classes, Grade I-IV, according to aggressiveness, anaplasia, and differentiation [1,2]. The primary challenge in treating GBM is its cellular heterogeneity, as the variation among cancer cells within the same tumor type leads to differential responses to treatments. Consequently, the existing treatment focuses on the differentiated cell population; nevertheless, this strategy has inadequately addressed GBM [3]. Similar to numerous other malignancies, NF-κB signaling is crucial to tumor proliferation and treatment resistance in GBM. The molecular mechanism responsible for the elevated activity of NF-κB in GBM has yet to be clarified [4]. This review examines the significance of nuclear factor-κB (NF-κB) signaling pathways in GBM and its interaction with other transcription factors, including FOSL1 and TRPM7, building upon our earlier study that investigated FOSL1’s involvement in GBM and associated pathways [2,5]. We conducted a search of electronic databases, including PubMed, Web of Science, and EMBASE. Inclusion criteria comprised peer-reviewed original articles and reviews published between 2010 and 2025 that focus on NF-κB signaling in GBM biology, glioma stem cells, tumor microenvironment, or therapeutic strategies. Excluded were articles not written in English, studies not directly related to GBM or NF-κB signaling, or papers without experimental or clinical relevance.

**Figure 1:**
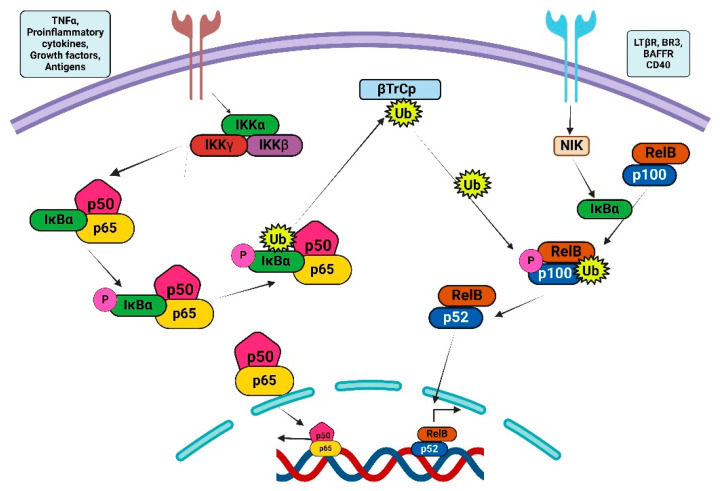
Illustrates the two principal routes implicated in NF-κB signaling. Canonical NF-κB signaling on the left side is mediated by the IKK complex, which comprises the IKKα, β, and γ subunits. The non-canonical NF-κB signaling pathway is mediated by IKKα homodimers.

NF-κB is integral to various biological functions, encompassing cell proliferation, inflammation, immune response, and apoptosis. The NF-κB family is characterized by the conserved N-terminal REL homology domain (RHD), which is essential for DNA binding and dimerization among NF-κB family members. The NF-κB complex has five subunits: RELA (p65), RELB, c-REL, p50, and p52. Among these, RELA, RELB, and c-REL possess an extra C-terminal transactivation domain (TAD). These molecules frequently function as heterodimers (e.g., p50:RELA) but occasionally also operate as homodimers (e.g., RelA) [6] (Figure 1).

Both signaling pathways and NF-κB subunits govern the expression of specific sets of target genes [7]. Given the strong association between NF-κB signaling, cytokine synthesis, and immunological responses, it was reasonable to investigate the role of NF-κB in hematopoietic cells. Reducing RelB in the germline resulted in the emergence of mice displaying myeloid hyperplasia and extramedullary hematopoiesis. The concurrent ablation of c-Rel and RelA led to cells exhibiting impaired engraftment and erythropoiesis, alongside unregulated proliferation of granulocytes [8]. These observations suggest diverse yet interrelated roles for distinct NF-κB subunits. Further investigations elaborated on these findings, suggesting that the extent of NF-κB activation is meticulously regulated in hematopoietic stem cells (HSCs) [9]. The elimination of RelA in HSCs results in changes in gene expression patterns that signify diminished HSC maintenance and homeostasis, alongside an increase in genes associated with lineage-specific cells. It was found that non-canonical NF-κB contributes to HSC self-renewal, influencing both intrinsic mechanisms and microenvironmental interactions. Certain studies suggest that the activation of NF-κB, whether by TLR activation or the lack of miR-146, enhances myeloid differentiation in HSCs [10,11,12]. A similar trend is observed in embryonic stem cells, where a minimal level of NF-κB activation is documented. Inhibiting NF-κB in these cells unexpectedly induces their differentiation. The elevated expression of p65 in embryonic stem cells resulted in enhanced differentiation and diminished pluripotency, underscoring the necessity of sustaining balanced NF-κB activity [13,14]. Research on muscle stem cells indicated a reduction in canonical NF-κB signaling during differentiation, followed by an activation of non-canonical NF-κB at a later stage, highlighting the unique functions of both pathways in stem cell biology [15]. This research collectively underscores the role of NF-κB signaling in maintaining diverse stem cell types, consistent with the current literature on NF-κB in cancer stem cells (CSCs).

Recent studies indicated a substantial role of nuclear factor kappa-light-chain-enhancer of activated B cells (NF-κB) signaling in GBM, highlighting NF-κB activation as a critical element contributing to the malignancy linked to poor prognoses in GBM patients [16]. The activation of NF-κB is a characteristic hallmark of inflammation and has garnered much focus regarding inflammation-induced malignancy. Evidence of inflammation in GBM is demonstrated by macrophage/microglia infiltration, lymphocyte presence, release of inflammatory cytokines, and activation of NF-κB, suggesting a possible involvement of inflammation in gliomagenesis. However, prominent signs of substantial inflammation are few in the majority of GBM cases, and NF-κB activation in GBM is probably due to genetic abnormalities and abnormal signaling [17,18]. NF-κB denotes a collection of transcription factors that attach to the enhancer region of the immunoglobulin kappa light chain in activated B cells [19]. NF-κB is structurally composed of homodimers and heterodimers created by the five constituents of the Rel family: NF-κB1 (p50/p105), NF-κB2 (p52/100), RelA (p65), and c-Rel. In the absence of stimulation, NF-κB remains inactive due to its association with the inhibitor IκBα and is predominantly situated in the cytoplasm. Upon exposure to stimuli like cytokines or DNA damage, IκB kinases, namely IKKα or IKKβ, are activated. This activation causes the phosphorylation of IκBα, which then undergoes degradation via a K48 ubiquitin-mediated proteasomal pathway [20]. The freed NF-κB subsequently translocates to the nucleus, where it acts as a transcription factor for many downstream target genes. Cytokines such as tumor necrosis factor alpha (TNFα), tumor necrosis factor-related apoptosis-inducing ligand (TRAIL), epidermal growth factor (EGF), and vascular endothelial growth factor (VEGF), along with DNA-damaging chemicals, can activate NF-κB via the canonical route. The activation of IKKs entails multiple upstream components, such as IKK gamma (NEMO), RIPK1, TAK1, TRAF1/2, and cIAP1/2. In the non-canonical NF-κB activation pathway, IKKα phosphorylates the p100 precursor, leading to the formation of a p52/RelB dimer that translocates to the nucleus to activate transcription [21].

## 2. NF-κB Signaling Pathway

The NF-κB signaling system is integral to numerous physiological processes, enabling cellular adaptation and response to external stimuli, and is essential for survival. Various environmental cues trigger the activation of NF-κB, which regulates gene expression that is crucial for immunological and stress responses. Various triggers initiate the activation of NF-κB, chiefly by phosphorylation by IKK and the subsequent destruction of IκB proteins. Upon release, NF-κB dimers translocate to the nucleus, regulating the transcription of several genes involved in cytokines, growth factors, cell adhesion molecules, and both pro- and anti-apoptotic proteins. The IKK complex consists of two closely similar kinase subunits, IKKα and IKKβ, plus a non-enzymatic regulatory component, IKKγ/NEMO [22]. Two mechanisms exist for the activation of NF-κB. The initial process, termed the classical pathway, is typically activated by exposure to proinflammatory cytokines or microbial and viral diseases. This activation entails the tripartite IKK complex, resulting in the phosphorylation-mediated destruction of IκB. The classical pathway largely influences p50:RelA and p50:c-Rel dimers and is predominantly dependent on the activity of IKKβ [23].

### Classical and Alternative NF-κB Signaling Pathways

The conventional mechanism that initiates NF-κB activation is mediated by IKK, resulting in the phosphorylation of IκB. This system reacts to multiple proinflammatory stimuli, including TNF, IL-1, lipopolysaccharide (LPS), and double-stranded RNA. In contrast, the alternate NF-κB activation pathway is facilitated by IKK, leading to the phosphorylation of p100 and its subsequent transformation into p52. The activation of IKK in this pathway is initiated by the upstream kinase NIK. The NIK/IKK/NF-κB2 pathway is activated upon engagement of the lymphotoxin receptor (LTR) in stromal nonlymphoid cells. This pathway is probably important for stimulating the synthesis of B-lymphocyte chemoattractant, an essential cytokine for adequate lymphoid organ development, as articulated by Ulrich Siebenlist (Bethesda) [24]. Furthermore, investigations performed in our group revealed that the NIK/IKK/NF-κB2 signaling pathway is activated in B cells via the interaction of the BAFF-R/BR-3 receptor with the B cell survival factor BlyS/BAFF. This conclusion is derived from the examination of bone marrow stem cells devoid of both NF-κB1 and NF-κB2, which were implanted into recipient animals lacking RAG. Despite the absence of evident irregularities in T cell development, a notable reduction in mature and late transitional B cells in the spleen was noted. Transitional B cells that had recently moved from the bone marrow to the spleen were predominantly unharmed [24,25]. The alternative pathway is essential for the survival of immature B cells and the development of secondary lymphoid organs. The anti-apoptotic role of the classical pathway mediated by IKKβ is crucial for multiple immunoreceptors, such as T and B cell receptors, TLR4, and the type 1 TNF-α receptor (TNFR1). These receptors provide both pro-survival and pro-apoptotic signals upon activation [26]. In the majority of circumstances, the signals that encourage cell survival dominate. Nonetheless, when IKKβ or NF-κB functions are impaired, receptor activation results in cellular apoptosis [27].

**Figure 2:**
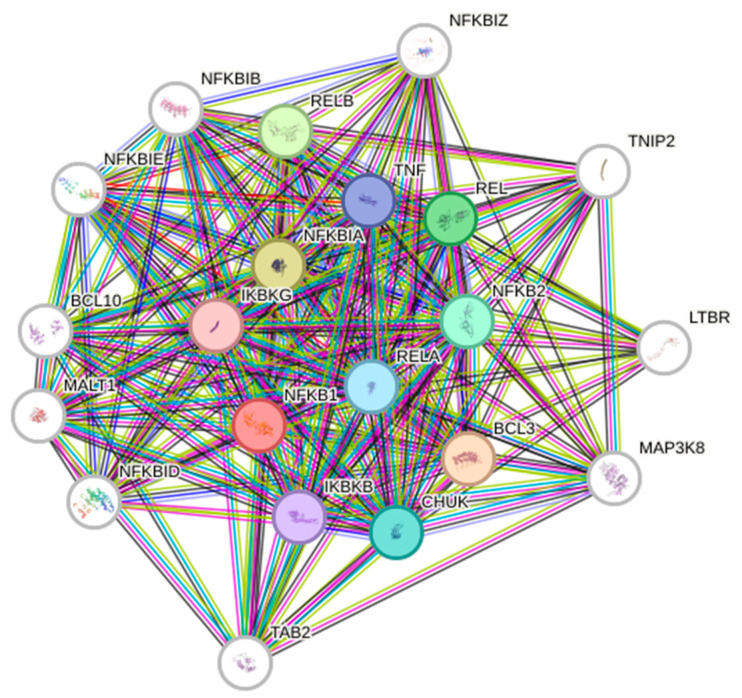
STRING database output of prominent NF-κB interaction members.

The non-canonical NF-κB signaling pathway originates with p100, which acts as a precursor to p52. P100 operates as an IκB molecule, inhibiting the nuclear translocation of RelB. The p100 activates p52 and stimulates the nuclear translocation of the RelB/p52 heterodimer. The co-translational processing of p105 and p100 is meticulously regulated by both inhibitory and stimulatory domains [28,29]. In numerous instances, p100 interacts with RelB, triggering its proteolytic activities, resulting in the development of the active RelB/p52 complex. An alternative mechanism linked to NF-κB activation is referred to as an unusual activation pathway. During genotoxic stress, the IKK complex is activated by ATM kinase, resulting in the ubiquitination of NEMO [20]. The activation of certain NF-κB dimers results in the localization of IKKs. Generally, they translocate into the nucleus, enabling Rel homology domains to associate freely with specific DNA sequences in the promoters of target genes. Depending on the cell type, several target genes may be transcriptionally activated, influenced by multiple additional variables that can either downregulate or upregulate NF-κB expression and interact with signaling pathways that can activate other transcription factors. The varied modifications in the post-translational of RelA introduce an additional layer of intricacy to NF-κB signaling. This has demonstrated the necessity of many RelA functions. Phosphorylation of serine or threonine residues is crucial for enhancing transcriptional activity. The activity of NF-κB is significantly affected by various phosphorylation events and protein–protein interactions, creating a complex network of interdependencies and feedback loops [30]. Moreover, additional members of the NF-κB family are actively involved in interactions with diverse inhibitors, including IkBα, IκBβ, or IκBϵ. These members also interact with upstream kinases and chromatin modifiers, including histone deacetylases (HDACs), p300, and other transcription factors [31]. The protein interaction networks are highly intricate; currently, there are 306 binary interactions of RelA documented in the IntAct interaction database (http://www.ebi.ac.uk/intact/ (accessed on 10 May 2025)). Figure 2 presents the STRING database output, showcasing both functional and physical interactors of the NF-κB proteins: RelA, RelB, NFκB1, and NFκB2.

## 3. NF-κB in GBM

The activation of NF-κB frequently transpires in several cancers. Deregulated NF-κB activation often aids oncogenesis by promoting tumor growth and invasion, suppressing apoptosis, and imparting treatment resistance [32]. Dysregulated NF-κB activity in cancer may result from various factors, including mutations or altered expression of genes encoding NF-κB proteins, or more frequently, disruptions in the regulatory mechanisms controlling NF-κB dimer activation [33]. Recent progress in GBM research has enabled the isolation of stem-like cell cultures from surgically removed brain tumors of specific individuals. These patient-derived cells exhibit stem-like properties in vitro and can sequentially form brain tumors when put into the cranial cavities of host mice under limiting dilution conditions. Glioblastoma patient-derived stem-like cells (GSCs) have the ability, similar to normal neural stem cells, to generate progeny cultures comprising both stem-like and highly differentiated non-stem-like descendants. This presents a previously unattainable chance to analyze the activity of several GBM cell types in mixed cultures derived from single stem-like cells collected from individual patients. This scenario provides a physiologically pertinent experimental framework to examine the mechanisms influencing the behavior of several GBM cell types implicated in tumor initiation, development, and recurrence [3,34].

The primary anomaly of NF-κB activation is frequently observed in GBM, with multiple pathways associated with the dysregulation of NF-κB signaling in gliomas. Epidermal growth factor receptor (EGFR) and platelet-derived growth factor receptor (PDGFR) exhibit aberrant activation and are interrelated with the activation of NF-κB expression via several mechanisms, encompassing both protein kinase B/AKT-dependent and independent pathways. The two oncogenic signaling systems are essential for various physiological activities, including cell proliferation, invasion, and tumor promotion through NF-κB expression [35,36].

Emerging evidence indicates that NF-κB signaling plays distinct and subtype-specific roles in GBM, with the most pronounced activation observed in the mesenchymal (MES) subtype. The MES GBM subtype, which is associated with a more aggressive phenotype and poorer prognosis, shows elevated expression of NF-κB target genes and upstream activators such as TNF-α, RELB, and TRADD, compared to other subtypes [37,38]. NF-κB acts as a lineage-defining transcription factor in MES GBM, where it cooperates with transcriptional regulators like STAT3, C/EBPβ, and FOSL1 to drive the mesenchymal gene expression program and promote inflammatory, invasive, and therapy-resistant features [39,40,41]. In contrast, the proneural (PN) subtype—characterized by PDGFRA amplification and higher expression of oligodendrocytic markers—exhibits relatively lower basal NF-κB activity, and its tumor biology is more influenced by pathways such as Notch and IDH1/2 mutations [42,43]. Interestingly, radiation or therapeutic stress can induce a PN-to-MES transition, partially mediated by NF-κB and STAT3 activation, suggesting that NF-κB not only marks a static subtype but may also drive dynamic Plasticity and treatment-induced reprogramming [44]. These observations underscore the importance of considering molecular subtype context when designing NF-κB-targeted therapies, as MES-specific NF-κB inhibition may offer the most benefit while sparing less NF-κB-dependent PN populations.

## 4. Role of Other Transcription Factors with NF-κB in GBM

**Figure 3:**
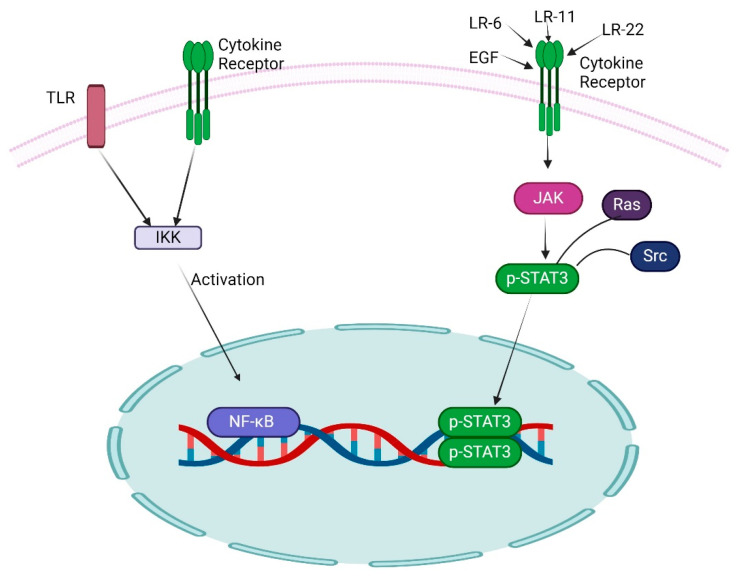
Illustrates the activation of NF-κB and STAT3. The quantity of external stimuli can activate NF-κB and STAT3 through IKK and JAK, respectively.

The signal transducer and activator of transcription 3 (STAT3) is a key member of the STAT family, notably participating in NF-κB transcription, and exists in the cytoplasm as an inactive form in non-stimulated cells. The activation of STAT3 is independent of the inducible degradation of any inhibitors. It effectively facilitated the phosphorylation of the critical amino acid residue Tyr 705, which starts the dimerization of STAT3 through phosphotyrosine-SH2 domain interaction [45]. Following dimerization, the STAT transcription factor translocated to the nucleus and activated a broad spectrum of target genes. Conversely, the unphosphorylated STAT3 can also undergo dimerization and activate transcription [46]. The JAK family of tyrosine kinases is a key mediator of STAT activation; for example, JAK1 significantly mediates STAT3 activation, and the transcriptional activity and DNA binding mechanism of STAT3 are augmented through Ser 727 phosphorylation (Figure 3). The reversible acetylation of STAT3 is an additional mechanism that augments STAT3 activity while simultaneously influencing the activity of NF-κB family members [47].

The inhibited function of STAT3 results in the irregularity of certain developmental processes and is also vital for homeostasis. STAT3 activity is extensively controlled by several feedback mechanisms, and its sustained activation results in malignancies [48]. The activation of STAT3 through feedback pathways is intricate, with SHP2 phosphate and the STAT3-inducible gene SOCS3 significantly contributing to the inhibition of STAT3 activation via the gp130 receptor [49]. STAT3 can associate with over 3000 gene promoters, encompassing the majority of NF-κB family members. STAT3 and NF-κB substantially regulate both unique and shared genes throughout carcinogenesis. This can be characterized by the distribution of NF-κB and the binding sites for STAT3; for example, one gene possesses solely NF-κB binding sites that respond to NF-κB, whereas another gene contains binding sites that may jointly regulate both factors [50].

## 5. Role of NF-κB in GSCs

In GBM, sustained and unregulated NF-κB activity is frequently noted, with many mechanisms suggested as potential reasons. An example is the continual activation of receptor tyrosine kinases, including the epidermal growth factor receptor (EGFR) and the platelet-derived growth factor receptor (PDGFR), in GBM. The activation of NF-κB in GBM entails both protein kinase B/AKT (AKT)-dependent and AKT-independent pathways, with the activation of EGFR and PDGFR serving as significant contributions. The molecular characteristics of GBM encompass EGFR gene amplification and the overexpression of a constitutively active version, which have been examined as possible treatment targets [51]. Moreover, cellular interaction mediated by IL-6 and NF-κB diminishes the effectiveness of EGFR tyrosine kinase inhibitors (TKIs). Targeting the NF-κB gene, IL-6 has been shown to markedly diminish the survival of GSCs in GBM, leading to a concurrent reduction in tumor growth [52]. In GBM, NF-κB may influence the control of pyruvate kinase M2 (PKM2), an enzyme that regulates the rate-limiting step of glycolysis [36].

A20 (TNFAIP3) was found to be higher in GSCs. This gene is known to be an NF-κB target gene and a cell survival regulator. Higher amounts of A20 in GSCs were linked to stronger resistance to apoptosis [53]. A recent genetic study intriguingly found the deletion of the IκBα (NF-KBIA) gene in around 24% of GBM patients [54]. Happold and his colleagues indicated that O6-methylguanine DNA methyltransferase (MGMT) functions as a positive regulator of NF-κB, mediated by NF-κB p65 in GSCs. Furthermore, another study has indicated that MGMT promoter hypermethylation is a significant biomarker and serves as a possible prognostic factor [55]. Attempts have been undertaken to classify GBM treatments based on the methylation state of MGMT [56]. The study also revealed the conversion of rat neurosphere cells into a growth factor-independent state through induced cultivation, characterized by persistently active NF-κB and the production of VEGF [57]. Under these discoveries, NF-κB in GBM is involved in the regulation of angiogenesis through VEGF mediation. Recent findings indicate that extracellular vesicles secreted by GSCs encompass VEGF-A [58,59]. The concept of employing VEGF receptor inhibition as a treatment approach for GBM has garnered significant interest in recent years. Bevacizumab, a VEGF inhibitor, has received FDA approval and is extensively employed in the United States. Nonetheless, several critics contend that although bevacizumab effectively diminishes peritumoral edema, it exhibits restricted tumoricidal efficacy, as seen by the absence of a definitive survival advantage in both initial and recurrent GBM patients across multiple extensive prospective trials [60].

GSCs demonstrate considerable resistance to Smac mimetics, a category of small-molecule pharmaceuticals that impede inhibitor of apoptosis (IAP) proteins. In GSCs, the injection of Smac mimetics triggers an adaptive response marked by increased production of TNFα and sustained activation of NF-κB and STAT3 signaling pathways. The interaction between NF-κB and STAT3 is associated with tumor progression and the promotion of stemness characteristics in certain cancers, including gliomas [61]. The interaction between NF-κB and STAT3 facilitates tumor progression and promotes the expression of stem-like traits in diverse malignancies, such as gliomas [62]. Furthermore, GSCs demonstrate a self-sustaining mechanism by releasing Sema3C while simultaneously expressing Plexin A2/D1 receptors. This initiates Rac1/NF-κB signaling in an autocrine/paracrine loop, enhancing their survival. A distinct mechanism contributing to NF-κB activation in glioblastoma is the TGF-β/TAK1 signaling axis, which can activate both canonical and non-canonical NF-κB pathways [63]. Proneural (PN) and mesenchymal (MES) GSCs are two separate, mutually exclusive subgroups that exhibit biological differences. Recent findings indicate that mixed lineage kinase 4 (MLK4) is expressed at higher levels in MES GSCs compared to PN GSCs. The inhibition of MLK4 led to the decrease in self-renewal, motility, tumorigenesis, and radio-resistance specifically in MES cells. MLK4 was identified as interacting with and phosphorylating IKKα, the regulator of NF-κB, hence initiating the activation of NF-κB signaling in MES [64]. The telomerase reverse transcriptase (TERT) has been newly discovered as a target gene of NF-κB. TERT may activate NF-κB p65 (RelA), establishing a positive feedback loop that amplifies the association with the newly found TERT promoter polymorphism. Mutations in the TERT promoter are thought to differentiate primary GBM from secondary GBM (which develops from a preexisting lower-grade glioma) and may act as a prognostic indicator [65,66]. Furthermore, TERT function has been shown to elicit features of GSCs via EGFR expression. The findings demonstrated that the IKK/NF-κB signaling pathway substantially contributes to the maintenance of GSCs.

The association between STAT3 and NF-κB indicates that NF-κB may transcriptionally modulate FOSL1 and have a role in gliomagenesis. To examine the downstream molecules of FOSL1, we investigated the transcriptome following the overexpression of FOSL1 in a PDX-L14 line known for its poor FOSL1 expression [67]. Subsequently, we performed immunohistochemistry staining for FOSL1 and NF-κB p65 utilizing rabbit polyclonal antibodies against FOSL1 and NF-κB p65 in glioma tissue microarrays (TMA) obtained from 141 glioma patients and 15 healthy controls. Subsequently, mutants of the human FOSL1 promoter, incorporating alterations in critical binding sites for NF-κB, were produced utilizing a Q5 site-directed mutagenesis kit. We subsequently analyzed luciferase activity in glioma cells and compared it to the wild-type FOSL1 promoter. Subsequently, we investigated the reciprocal regulation between NF-κB signaling and FOSL1 by altering the expression levels of NF-κB or FOSL1. Thereafter, we evaluated the activity of FOSL1 and NF-κB. To elucidate the function of FOSL1 in cellular proliferation and stemness, we performed a CCK-8 experiment and cell cycle analysis, evaluating apoptosis and GSC markers, ALDH1 and CD133, under different FOSL1 expression levels. Transcriptomic studies of downstream components of FOSL1 indicate that the NF-κB signaling pathway is modulated by FOSL1. The expression of NF-κB p65 protein is correlated with FOSL1 expression in glioma patients, and both are linked to glioma grades. NF-κB is an essential transcription factor that activates the FOSL1 promoter in glioma cells. The reciprocal control of NF-κB and FOSL1 facilitates glioma carcinogenesis and stemness by enhancing the G1/S transition and suppressing apoptosis. Consequently, the FOSL1 molecular pathway is functionally linked to NF-κB activation, promotes stemness, and suggests that FOSL1 could serve as a novel therapeutic target for GBM.

## 6. The Reciprocal Influence Between NF-κB and Non-Coding RNA (ncRNAs)

MicroRNAs, measuring 21 to 25 nucleotides in length, represent a class of non-coding, single-stranded RNA molecules. They perform essential roles in the regulation of post-transcriptional gene expression. As of now, about 2000 microRNAs have been identified in the human genome. The majority of miRNA genes are located within introns or exons, present in both coding and non-coding transcription units, with a considerable fraction arranged in clusters [68]. RNA polymerase II (Pol II) is essential for transcribing the majority of miRNA genes, hence commencing the synthesis of primordial miRNA (pri-miRNA). Thereafter, the Drosha enzyme, in conjunction with its cofactor provided by the DiGeorge syndrome critical region gene 8 (DGCR8), cleaves the pri-miRNA, producing precursor miRNA (pre-miRNA), a stem-loop structure around 70–80 nucleotides in length. The pre-miRNA hairpin is then transferred from the nucleus to the cytoplasm by exportin-5. Upon entering the cytoplasm, the stem loop is broken by the RNAse III enzyme Dicer, resulting in the formation of double-stranded miRNA. In malignancies, aberrant miRNA expression is a characteristic event resulting from the amplification, deletion, and translocation of miRNA genes, as well as the dysregulation of transcription factors such as p53 and c-Myc. The upregulated miRNAs act as oncogenes (oncomiRs), whereas the downregulated miRNAs function as tumor suppressors [69,70]. Table 1 shows the major miRNA that are involved in GBM and targets NF-κB pathway.

The expression and roles of miR-451 in GBM remain disputed. Although miR-451 expression is suppressed in GBM, where it acts as a tumor suppressor, contradictory studies suggest that miR-451 significantly enhances GSCs. The contradictory data suggest that miR-451 is regulated as a metabolic adaptation. Elevated miR-451 correlates with the suppression of the CAB39/LIKB1/AMPK pathway, which activates mTOR and facilitates cell proliferation; this mechanism suggests a shift from migration to proliferation in GBM cells. Elevated miRNAs are prevalent in physiological fluids, including plasma and cerebrospinal fluid (CSF), and engage in several intracellular interactions, hence functioning as diagnostic biomarkers [96]. Van der Vos et al. showed that miR-21 and miR-451 were overexpressed in extracellular vesicles produced from two patient-derived glioblastoma cell lines (GBM11/5 and GBM20/3) [97]. Furthermore, the microglial cells exhibited a notable elevation in miR-451. Gene and protein expression analyses revealed a reduction in the expression of the target genes PTEN (miR-21) and MIF (miR-451). Additionally, both miR-21 and miR-451 displayed a synergistic effect in inhibiting the activity of c-Myc [98]. Jacob et al. utilized 14 patient-derived and four commercially accessible cell lines to ascertain that miR-194-3p serves a pivotal function as an epigenetic factor in defining stemness and transcriptional subtype in GBM [99]. They present evidence that miR-194-3p induces the degradation of TAB2, an essential modulator of NF-κB activity, leading to a decrease in NF-κB transcriptional activity. The overexpression of miR-194-3p or the silencing of TAB2 led to a reduction in NF-κB activity. Because NF-κB activity went down, iPSC genes were expressed less, the oncogenic IL-6/STAT3 signaling pathway was blocked, the mesenchymal transcriptional subtype was slowed down in favor of the proneural subtype, and differentiation from GSCs to a monolayer phenotype was sped up. Targeting the miR-194-3p/TAB2/NF-κB signaling axis may be a way to reduce gene expression variations in GBMs because it acts as an epigenetic switch that controls the plasticity of GBM. The therapeutic role of non-coding RNAs (ncRNAs) in GBM is an increasingly promising area of research. ncRNAs are involved in regulating gene expression and play critical roles in GBM pathogenesis, progression, and treatment resistance. Their dysregulation contributes to multiple hallmarks of GBM, including proliferation, stemness, invasion, angiogenesis, and immune evasion. Non-coding RNAs represent a novel class of therapeutic targets and agents in glioblastoma. Targeting or restoring ncRNAs can modulate key signaling pathways, reverse drug resistance, and inhibit tumor progression. Though still in early clinical translation, advances in delivery systems and molecular design are expected to pave the way for ncRNA-based GBM therapies.

## 7. Role of NF-κB Signaling in Tumor Microenvironment

The tumor microenvironment (TME) consists of tumor cells, immune cells, various stromal cells, and mediators such as chemokines and cytokines, including endothelial cells, myeloid-derived suppressor cells (MDSCs), and cancer-associated fibroblasts (CAFs), all of which are essential for the TME [100]. NF-κB is integral in various cell types for modulating the tumor microenvironment and responding to external stimuli, such as TLR ligands. TNFα can directly influence the transition of macrophages to the M1 phenotype, typically exhibiting tumor-suppressive properties. However, in different contexts, NF-κB activation can trigger the transcription of numerous genes associated with M2 polarization, thereby facilitating tumor growth. Cytokines are soluble molecules generated by cancer cells, stromal cells, and immune cells that significantly contribute to metastasis and inflammation; therefore, comprehending the mechanisms and relationships between NF-κB and pro-tumorigenic cytokines is essential. Tumor necrosis factor α (TNFα) is a prominent tumor-promoting cytokine that facilitates carcinogenesis across several cancer types [101]. Macrophages and neutrophils primarily contribute to the synthesis of TNFα, which subsequently stimulates the production of other proinflammatory cytokines, including IL-6 and IL-1β, thus promoting cancer. IL-6 is a significant and abundant cytokine in the tumor microenvironment, produced by malignant tumor cells that stimulate cancer-related inflammation and promote stem cell proliferation [102]. Another significant cytokine is IL1α/β, synthesized by cancer and immune cells, which are heavily reliant on NF-κB, and stimulate the MAPK and NF-κB pathways, contributing to tumorigenesis [103]. IL-33, a member of the IL-1 family, is prominently produced in tumor cells as well as in epithelial and fibroblast cells, and it plays a pivotal role in autoimmunity, allergies, cancer, and inflammation. TGF-β is a significant cytokine produced by various cells, including fibroblasts, T cells, and cancer cells, which are vital for cell differentiation and serve as potent tumor inducers, moreover playing a critical role in tumor invasion and metastasis [104].

NF-κB modulates chemokine expression in tumor cells inside the tumor microenvironment, especially in CAFs. The increase in NF-κB and other transcription factors, including AP1 and STAT3, enhances the expression of chemokines essential for immune cells, hence intensifying the inflammatory response and facilitating the proliferation and dissemination of primary tumors [105]. The function of chemokines in carcinogenesis is intricate and unclear. The chemokine (C-C motif) ligand CCL5 induces the expression of the eukaryotic initiation factor 4F translation initiation complex in a mechanistic target of rapamycin (mTOR)-dependent manner, and enhances the upregulation of cyclin D1, c-Myc, and Dad-1 protein expression, consequently promoting cell proliferation [106].

The inflammatory cells demonstrate substantial production of tumor-associated cytokines, encompassing tumor-associated macrophages (TAMs) and neutrophils. The activation of NF-κB is significantly increased in these cells, resulting in the secretion of cytokines such as IL6, TNFα, and IL1β, which subsequently promote cell proliferation and tumor viability [107]. In the tumor microenvironment, macrophages are prevalent and release inflammatory cytokines and chemokines, thereby promoting the production of cysteine cathepsin, which is essential for tumor growth [100]. Macrophages operate in two distinct contexts within the tumor microenvironment: M1 macrophages, activated by INFγ, secrete proinflammatory chemokines and enzymes vital for inflammation, whereas M2 macrophages, stimulated by IL4 and IL-10, can suppress mediators and contribute to an immunosuppressive tumor microenvironment. The heightened activity of p50 affects the polarization of M1 and M2 macrophages. The inhibited activity of NF-κB induces the transformation of M2 macrophages into M1 macrophages, as this polarization necessitates IL-IR and MyD88, while M2 macrophages depend on IKKβ-mediated activation of NF-κB [108]. Furthermore, the compromised NF-κB activation in tumor-associated macrophages enhances the tumoricidal action of macrophages. Suppressed NF-κB activity results in the M1 phenotype, characterized by anti-tumor cytotoxicity; hence, NF-κB may serve as a viable target for modulating macrophage phenotype within the tumor microenvironment [109,110].

Dendritic cells (DCs) are crucial in the signal inhibition mediated by immunological checkpoint molecules, particularly programmed cell death protein 1 (PD1), which substantially suppresses cytokine production. Conversely, the ligand of programmed cell death 1 ligand 1 (PD-L1) functions as a target for NF-κB genes. The augmented activity of PD-L1 in neoplastic cells induces NF-κB activation by many stimuli and activators, including oncogenes, cytokines, stressors, and chemotherapeutic agents [109]. Natural killer cells are essential in the destruction of cancer cells and exhibit anticancer activity; the activation of cytotoxic effector molecules, including perforin and granzyme B, is also regulated by NF-κB. The augmented activity of NF-κB in natural killer cells can be induced by anticancer agents such as paclitaxel [111]. T and B cells are crucial elements in the tumor microenvironment, strongly influencing tumor promotion or anti-cancer activity. The activation of conventional T cells is essential for the engagement of canonical NF-κB pathways, which are critical for CD8+ T cell proliferation and the start of an anti-tumor immune response. The activation of Treg cells requires the participation of p65 and Rel, which are essential for maintaining optimal homeostasis, maturation, and the preservation of immunological tolerance in Treg cells [112,113]. In B cells, IKKβ-dependent NF-κB is activated to produce the cytokine lymphotoxin (LT), a heterotrimeric component of the TNF family essential for IKKα activation [114]. The manufacture of LT in B cells activates IKKα, which phosphorylates a site on the transcription factor E2F1, enabling its translocation and recruitment to the Bmi1 gene target [115].

Fibroblasts are a crucial component of the tumor microenvironment. The standard fibroblast experiences intrinsic modifications and, in hypoxic environments, converts into cancer-associated fibroblasts (CAFs), demonstrating increased production of several inflammatory factors and chemokines [116]. IL-1β stimulates the NF-κB pathway, promoting cell proliferation, angiogenesis, and metastasis. The findings indicate that the complex function of NF-κB in various cells of the tumor microenvironment is a viable target for pharmacological intervention.

## 8. NF-κB Inhibitors in GBM: Utilization and Insights from Clinical Trial Studies

NF-κB is a prominent signaling pathway linked to immune response; nonetheless, it frequently exhibits dysregulated signaling in GBM. Aberrant signaling has been linked to many tumor-related behaviors, including cancer cell proliferation, invasiveness, and heightened resistance to chemotherapy. Various stress events, such as reactive oxygen species (ROS), DNA damage, and growth factors, induce NF-κB activation in cancer [117]. Currently, most non-specific medications have been successfully employed for NF-κB targeting in preclinical GBM models. Table 2 shows NF-κB Inhibitors in GBM Clinical Trials.

Sulfasalazine is an IκK inhibitor frequently utilized in the treatment of rheumatoid arthritis. In Phases 1 and 2 of the study, sulfasalazine was given to 10 patients with advancing malignant glioma at four different dosages; however, no clinical response was observed, resulting in a median progression-free survival of 32 days [118]. The promising preclinical results with bortezomib were utilized in patients with newly diagnosed and recurrent GBM. In the Phase 2 research, bortezomib plus vorinostat were given to 37 patients with recurrent GBM, yielding a median overall survival of only 3.2 months. Furthermore, the Phase 1 trial demonstrated that the combination of temozolomide (TMZ)/IR with bortezomib was well tolerated [119].

**Figure 4:**
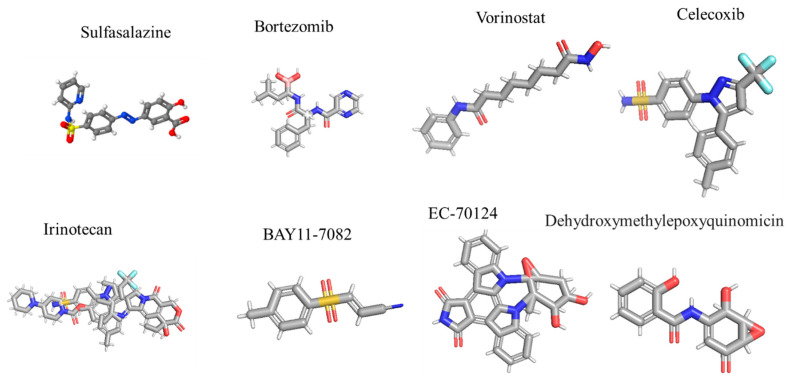
Represents the 3-D structure of currently used NF-κB inhibitors against GBM.

Celecoxib is employed in various clinical trials for the management of malignant glioma [120,121]. In the Phase 2 experiment, CPT-11 (irinotecan) and celecoxib were given to 37 patients with grade III and IV glioma, resulting in a median overall survival of 31.5 weeks [120]. In a distinct study, 50 patients with newly diagnosed glioblastoma multiforme, who underwent radiotherapy without prior chemotherapy, received temozolomide, celecoxib, and thalidomide. The results revealed a median overall survival rate of 12.6 months [124]. Multiple studies have demonstrated that IκK is crucial for NF-κB activation, consequently identifying IκK as a significant target for pharmacological intervention. Numerous IκK inhibitors have been employed in the management of peripheral cancer, although only a limited number have been utilized in the therapy of GBM. BAY11-7082 (Figure 4) is among the most utilized pharmaceuticals demonstrating considerable anti-glioma activity. The majority of NF-κB inhibitors were observed to enhance the sensitivity of GBM cells to doxorubicin and cisplatin, proving effective against chemotherapy-resistant clones. Another report indicated that the activity of a novel glycosylated indolocarbazole, EC-70124, significantly enhanced sensitivity in GBM stem-like cells. This report suggests that IκK inhibition may modulate other signaling pathways, including highly selective agents such as BAY11-7082; thus, this effect may not be exclusively attributable to NF-κB inhibition [122].

Another element of the NF-κB family, IκKβ, phosphorylates p53, IκKα, tuberous sclerosis 1, and FOXO3, which can translocate into the nucleus and interact with TGFβ-regulated Smad2/3. These results indicated that the inhibition of IκK can result in numerous NF-κB independent actions that are unrelated to off-target effects of particular inhibitors [125]. The unexpected behavior of the IκK complex renders it highly intriguing. However, owing to the heterogeneous nature of GBM, targeting IκK may influence several signaling pathways and potentially have significant inhibitory effects. Generally, the most successful approach for targeting NF-κB suppression involves blocking other subunits of NF-κB. Inhibition of p65, a modulator of NF-κB signaling, induces cytotoxicity in GBM cells, whereas the expression of p65 shRNA enhances GBM xenograft growth and vascular density [33]. Inhibiting the expression of intracellular antibodies (intrabodies) is an alternative approach to selectively obstruct protein expression. The activation of a single-chain intrabody targeting p65 in GBM cells markedly reduces NF-κB-dependent gene expression and diminishes intracranial xenograft proliferation [126]. Dehydroxymethylepoxyquinomicin (DHMEQ), a tiny chemical, selectively binds to cysteine residues in p64 and other Rel homologous proteins, markedly inhibiting the nuclear translocation of NF-κB and its DNA binding activity. In GBM, DHMEQ reduces cell growth and enhances animal survival by inhibiting NF-κB activation [123]. While the gold standard therapy for GBM, consisting of TMZ and NF-κB BAY11-7082, demonstrates enhanced apoptosis in patient-derived GBM cell lines, it also exhibits reduced migration. The initial studies indicated that these medications, when co-administered, may affect the crosstalk between phosphatidylinositol-3-kinase (PI3K), AKT, mTOR, and NF-κB [127].

Targeting NF-κB in GBM presents significant therapeutic challenges due to potential off-target effects and the activation of compensatory signaling pathways. Pharmacological inhibitors of NF-κB, such as proteasome inhibitors (e.g., bortezomib) or IKKβ inhibitors, can affect multiple cell types and interfere with essential immune functions, potentially leading to systemic immunosuppression and toxicity [128,129]. Moreover, GBM cells often exhibit adaptive resistance through the upregulation of alternative oncogenic pathways, including STAT3, PI3K/AKT/mTOR, and MAPK signaling, which can functionally compensate for NF-κB inhibition and maintain tumor survival and proliferation [130,131,132]. For instance, dual inhibition of NF-κB and STAT3 has shown synergistic effects in preclinical models, underscoring the interdependence and redundancy of these pathways [133]. Additionally, evidence suggests that chronic NF-κB inhibition may lead to epigenetic reprogramming and selection for more aggressive, therapy-resistant tumor cell subpopulations [134]. These findings highlight the need for precision-based combinatorial approaches and a deeper understanding of context-specific NF-κB signaling dependencies in GBM.

### Natural Compounds Against NF-κB in GBM

Resveratrol is a polyphenolic molecule that exhibits considerable inhibitory effects on invasion, immunomodulation, and the proliferation of cancer cells [135]. Resveratrol significantly inhibits the PI3K/AKT/NF-κB signaling pathway, resulting in the suppression of matrix metalloproteinases, which consequently hinders cell proliferation in GBM [136]. The research further validated the association between NF-κB activity and the invasiveness of GBM, facilitating the direct incorporation of metalloproteinases (MMPs) into adjacent tumor cells. Furthermore, resveratrol mitigated temozolomide resistance through the MGMT route downstream of NF-κB [137]. Quercetin is a prominent anticancer agent that successfully promotes apoptosis in breast, liver, and brain cancer cells. Quercetin markedly induces apoptosis in GBM cells by activating caspase-3 and facilitating NF-κB translocation into the nucleus [138]. Isothiocyanates are natural compounds obtained from cruciferous vegetables that demonstrate anti-invasion, anticancer, and anti-inflammatory properties [139]. In GBM, the conjunction of isothiocyanate and temozolomide activates the NF-κB dependent signaling cascade, therefore diminishing the expression of MGMT. In GBM cells such as U87, T98, and U373, the isothiocyanate enhanced the susceptibility to temozolomide resistance by inhibiting the expression of MGMT through the NF-κB signaling pathway [140]. A separate study indicated that isothiocyanate modulates MMP-9 expression in C6 glioma cells, which are essential for cancer spread that degrades the extracellular matrix. This work demonstrated that isothiocyanate inhibits the production of MMP-9 via suppressing NF-κB and activator protein-1 (AP-1), hence obstructing motility and invasion in C6 GBM cells [141].

**Figure 5:**
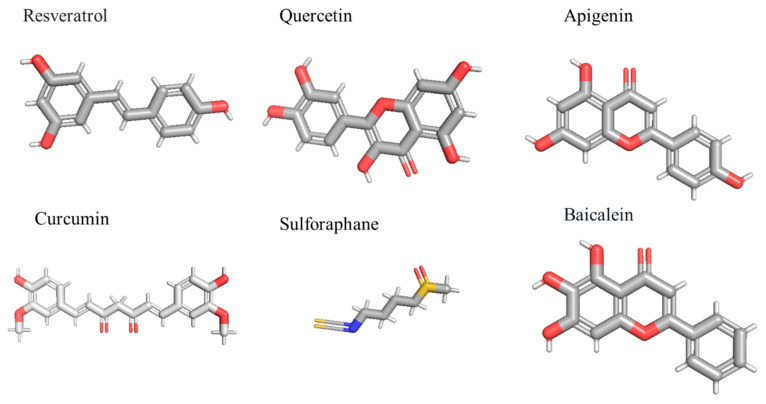
Represents the 3-D structure of plant-derived compounds that are used as NF-κB inhibitors against GBM.

Sulforaphane, obtained from broccoli sprouts, is a class of organosulfur compounds that exhibits considerable inhibitory effects on human cancer cells. Sulforaphane suppresses NF-κB-regulated gene expression through the IkBα and IκK signaling pathways [142]. It modified caspase 9/12 cleavage, intracellular Ca2+ concentration, cytochrome C release, and IkBα expression in glioma cell lines, including U87MG and T98G [143]. In GBM cells, sulforaphane markedly inhibits anti-apoptotic proteins and modulates IκBα, which subsequently results in the inhibition of NF-κB production [137]. A separate study indicated that sulforaphane (Figure 5) markedly inhibits the proliferation of temozolomide-resistant GBM cells, suppresses the NF-κB pathway, and subsequently reduces MGMT expression, thereby reversing chemoresistance to temozolomide in GBM cell lines, including U373-R, U87-R, and T98G. The conjunction of temozolomide and sulforaphane markedly enhances apoptosis and diminishes survival potential in temozolomide-resistant GBM cells [144]. Sulforaphane inhibits cell invasion by activating the ERK1/2 signaling pathway in GBM cells and induces apoptosis. Curcumin is a prominent bioactive molecule extracted from Curcuma longa L., exhibiting substantial anti-inflammatory and antioxidant effects by reducing AP-1 and NF-κB activity [145]. Due to their effectiveness in treating many high-risk malignancies, they have also been evaluated in Phase I clinical trials. Curcumin exerts substantial effects on GBM cells, inhibiting cell proliferation, invasion, and angiogenesis. In GBM cells, curcumin reduces cell viability in a manner that is independent of p53 and caspase, associated with the inhibition of the NF-κB and AP-1 signaling pathways [146].

## 9. Perspective

NF-κB is a crucial transcription factor that significantly influences various processes, including cancer, metastasis, angiogenesis, and drug resistance. Activation of NF-κB is driven by several causes, with increased NF-κB expression significantly contributing to cancer progression. Consequently, the suppression of NF-κB expression has emerged as a significant and appealing molecular target for cancer prevention and therapy [147].

NF-κB can be activated by various stimuli and intricate signaling pathways, which interact with one another. Over the past two years, we have acquired a deeper comprehension of NF-κB signaling in GBM, namely its involvement in inflammation, proliferation, migration, immune evasion, treatment resistance, and apoptosis [4]. A considerable amount of progress in tackling GBM has been accomplished via the manipulation of the EGFR/PI3K/AKT/ERK/NF-κB signaling pathways. The NF-κB signaling pathway is chiefly characterized by the phosphorylation, stability, and nuclear translocation of p65. This underscores our growing expertise in the signaling network that regulates GBM proliferation via NF-κB signaling. This review analyzes the ability of the NF-κB gene to influence GBM development, migration, and invasion. The primary anomaly of NF-κB activation is frequently observed in GBM, with multiple pathways associated with the dysregulation of NF-κB signaling in gliomas. What distinguishes NF-κB from other signaling hubs is its dynamic crosstalk with transcriptional regulators, metabolic networks, and non-coding RNAs (ncRNAs), which collectively fine-tune gene expression in response to environmental and intrinsic stimuli. The recent discovery of ncRNA-mediated regulatory feedback loops and chromatin-based NF-κB control mechanisms underscores the transcription factor’s versatility and potential as a therapeutic target. However, this complexity also poses challenges, as broad inhibition of NF-κB risks unintended immunosuppression and toxicity. Moreover, they offer inadequate clarification regarding the mechanisms through which the activation of the NF-κB pathway promotes cell proliferation, migration, invasion, immune evasion, and drug resistance, along with the justification for the induction of cell death following the inhibition of the NF-κB pathway. Consequently, additional research should concentrate on enhancing the rigor of experiments and elucidating the expression and molecular regulatory networks of downstream target genes within the NF-κB signaling cascade. This will clarify the mechanisms behind the proliferation, migration, invasion, immune evasion, and treatment resistance of GBM cells [148]. The fundamental importance of elucidating signal transduction pathways transcends understanding the chemical foundations of carcinogenic effects; it lies in their therapeutic application. Consequently, the NF-κB signaling pathway represents a viable target for therapeutic interventions. To date, research investigating NF-κB as a prospective therapeutic target has predominantly focused on nonspecific drugs or on targets such as IκK, which affect several signaling pathways. Despite decades of research, GBM remains an incurable malignancy with limited therapeutic options and a dismal prognosis. The persistence of therapy-resistant GSCs, profound intratumoral heterogeneity, and a highly immunosuppressive TME presents major barriers to effective treatment. Within this complex landscape, NF-κB signaling emerges as a central regulatory node—integrating diverse oncogenic cues and orchestrating transcriptional programs that sustain tumor growth, plasticity, and immune evasion. Future research must focus on achieving context-specific modulation of NF-κB activity. This includes deciphering the precise mechanisms by which NF-κB differentially governs GSC identity versus immune suppression, identifying biomarkers that predict NF-κB dependency in GBM subtypes, and leveraging single-cell and spatial transcriptomic technologies to uncover cell-type-specific NF-κB circuits. Additionally, combinatorial strategies that selectively target NF-κB alongside GSC regulators, immunomodulators, or metabolic pathways may offer a path toward overcoming resistance and enhancing long-term therapeutic responses.

In conclusion, the pivotal function of the NF-κB pathway in GBM proliferation and therapeutic resistance suggests that a comprehensive understanding of the regulation of this transcription factor in GBM could markedly enhance the effective management of these tumors.

## Figures and Tables

**Table 1:** Shows the major miRNA that are involved in GBM and targets NF-κB pathway.

S. No	miRNA	Target Pathway	Type of Diseases
1.	miR-146	TLR/NF-κB pathway	Breast cancer [71], pancreatic cancer [72]
2.	miR-155	Stimulates NF-κB, IKKε, Ripk1	Breast [73], Colon [74,75], Pancreas Cancer [76], osteosarcoma [77], GBM [78]
3.	miR-181b-1	STAT3, NF-κB	Glioma cells and astrocytic tumors [79]
4.	miR-21	NF-κB, PTEN	Lung cancer [80], chronic lymphocytic leukemia, acute myeloid leukemia and Hodgkin lymphoma [81], glioma [82] and GBM [83]
5.	miR-301a	NF-κB	Lung cancer [84], pancreatic tumor [85,86]
6.	miR-204-5p	TRAF1, MAP3K3, and TAB3 via inhibiting NF-κB	Prostate cancer [87]
7.	miR-18a	modulating the TNF-α-mediated NF-κB	Glioma [88]
8.	miR-650	targeting RERG via AKT/ERK/NF-κB pathways	Glioma [89]
9.	miR-30a-5p	targeting WWP1 via up-regulating NF-κB	Glioma [90]
10.	miR-129-5p	targeting Wnt5a via blocking JNK and PKC/ERK/NF-κB pathways	GBM [91]
11.	MicroRNA-30e	NF-κB/IκBα	Glioma [92]
12.	miR-182	NF-κB	Glioma [93]
13.	miR-221 and miR-222	NF-κB/p27Kip1	Colorectal cancer [94], glioma [95]

**Table 2:** NF-κB Inhibitors in GBM Clinical Trials.

Drug	Target/Mechanism	Trial Phase	Key Findings	Model/Cell Line	Reference(s)
**Sulfasalazine**	IKK inhibitor (blocks IκB degradation)	Phase I/II	No clinical response; median PFS: 32 days.	Recurrent GBM patients	[118]
**Bortezomib**	Proteasome inhibitor (blocks NF-κB activation)	Phase II	Combined with vorinostat: median OS = 3.2 months. TMZ synergy in Phase I.	U87MG, T98G, patient-derived GSCs	[119]
**Celecoxib**	COX-2 inhibitor (indirect NF-κB suppression)	Phase II	With irinotecan: median OS = 31.5 weeks. Reduced edema but limited survival benefit.	U251, LN229, patient tissues	[120,121]
**BAY11-7082**	IKKβ inhibitor (prevents IκBα phosphorylation)	Preclinical	Enhanced TMZ sensitivity; reduced migration in GBM cells.	U87, T98G, GSC xenografts	[122]
**DHMEQ**	Blocks NF-κB nuclear translocation	Preclinical	Suppressed GSC tumorigenicity; improved survival in murine models.	Patient-derived GSCs	[123]

## Data Availability

The data produced during the study are governed by a data-sharing requirement and are accessible in a public repository that does not assign DOIs to datasets.

## Abbreviations

AP-1	Activator protein-1
CAFs	Cancer-associated fibroblast
CSCs	Cancer stem cells
CSF	Cerebrospinal fluid
DCs	Dendric cells
DGCR8	DiGeorge syndrome critical region gene 8
DHMEQ	Dehydroxymethylepoxyquinomicin
E2F1	E2F transcription factor 1
EGF	Epidermal growth factor
EGFR	Epidermal growth factor receptor
GBM	Glioblastoma multiforme
GSCs	Glioblastoma stem cells
HDAC	Histone deacetylase
HSCs	Hematopoietic stem cells
IKK	IκB kinase
Intrabodies	Intracellular antibodies
LPS	Lipopolysaccharide
LTR	Lymphotoxin receptor
MDSCs	Myeloid-derived suppressor cells
MES	Mesenchymal
MGMT	O6-methylguanine DNA methyltransferase
MLK4	Mixed lineage kinase 4
MMPs	Metalloproteinases
ncRNAs	Non-coding RNA
NF-κB	Nuclear factor-κB
PD1	Programmed cell death protein 1
PDGFR	Platelet-derived growth factor receptor
PD-L1	Programmed cell death 1 ligand 1
PI3K	Phosphatidylinositol-3-kinase
PN	Proneural
Pol II	Polymerase II
RHD	REL homology domain
STAT3	Signal transducer and activator of transcription 3
ROS	Reactive oxygen species
TAD	Transactivation domain
TAMs	Tumor-associated macrophages
TERT	Telomerase reverse transcriptase
TME	Tumor microenvironment
TMZ	Temozolomide
TNFα	Tumor necrosis factor α
TRAIL	Tumor necrosis factor-related apoptosis-inducing ligand
VEGF	Vascular endothelial growth factor
